# South Asian Heart Risk Assessment (SAHARA): Randomized Controlled Trial Design and Pilot Study

**DOI:** 10.2196/resprot.2621

**Published:** 2013-08-20

**Authors:** Zainab Samaan, Karleen M Schulze, Catherine Middleton, Jane Irvine, Phillip Joseph, Andrew Mente, Baiju R Shah, Guillaume Pare, Dipika Desai, Sonia S Anand, SAHARA Investigators

**Affiliations:** ^1^Department of Psychiatry and Behavioral NeurosciencesMcMaster UniversityHamilton, ONCanada; ^2^Population Genomics Program, Chanchlani Research CentreMcMaster UniversityHamilton, ONCanada; ^3^Department of Clinical Epidemiology and BiostatisticsMcMaster UniversityHamilton, ONCanada; ^4^Population Health Research InstituteMcMaster University and Hamilton Health SciencesHamilton, ONCanada; ^5^Ryerson UniversityToronto, ONCanada; ^6^York UniversityToronto, ONCanada; ^7^Evaluative Clinical Sciences, Schulich Heart Research Program, Sunnybrook Research InstituteUniversity of TorontoToronto, ONCanada; ^8^McMaster UniversityPathology and Molecular MedicineHamilton, ONCanada; ^9^Department of MedicineMcMaster UniversityHamilton, ONCanada

**Keywords:** multimedia, South Asians, health, risk, assessment, randomized, trial

## Abstract

**Background:**

People of South Asian origin suffer a high burden of premature myocardial infarction (MI). South Asians form a growing proportion of the Canadian population and preventive strategies to mitigate the risk of MI in this group are needed. Prior studies have shown that multimedia interventions are effective and feasible in inducing health behavior changes among the obese, smokers, and among those who are sedentary.

**Objective:**

Among at-risk South Asians living in Canada, our objectives are to determine: (1) the feasibility of a culturally tailored multimedia intervention to induce positive behavioral changes associated with reduced MI risk factors, and (2) the effectiveness and acceptability of information communicated by individualized MI and genetic risk score (GRS) reports.

**Methods:**

The South Asian HeArt Risk Assessment (SAHARA) pilot study enrolled 367 individuals of South Asian origin recruited from places of worship and community centers in Ontario, Canada. MI risk factors including the 9p21 genetic variant status were provided to all participants after the baseline visit. Participants were randomly allocated to receive a multimedia intervention or control. The intervention group selected health goals and received personalized health messages to promote adherence to their selected goals. After 6 months, all participants had their MI risk factors repeated. The methods and results of this study are reported based on the CONSORT-EHEALTH guidelines.

**Results:**

The mean age of participants was 53.8 years (SD 11.4), 52.0% (191/367) were women, and 97.5% (358/367) were immigrants to Canada. The mean INTERHEART risk score was 13.0 (SD 5.8) and 73.3% (269/367) had one or two copies of the risk allele for the 9p21 genetic variant. Both the intervention and control groups made some progress in health behavior changes related to diet and physical activity over 6 months. Participants reported that their risk score reports motivated behavioral changes, although half of the participants could not recall their risk scores at the end of study evaluation. Some components of the multimedia intervention were not widely used such as logging onto the website to set new health goals, and participants requested having more personal interactions with the study team.

**Conclusions:**

Some, but not all, components of the multimedia intervention are feasible and have the potential to induce positive health behavior changes. MI and GRS reports are desired by participants although their impact on inducing sustained health behavior change requires further evaluation. Information generated from this pilot study has directly informed the design of another randomized trial designed to reduce MI risk among South Asians.

**Trial Registration:**

ClinicalTrials.gov NCT01577719; http://clinicaltrials.gov/ct2/show/NCT01577719 (Archived by WebCite at http://www.webcitation.org/6J11uYXgJ).

## Introduction

### Background

Myocardial infarction (MI) due to coronary artery disease (CAD) remains a major cause of death globally [1]. The rising prevalence of overweight, obesity, and type 2 diabetes is predicted to potentiate the CAD epidemic in developing countries [2]. South Asians, people who originate from the Indian subcontinent, suffer a high burden of premature MI [3,4], and are projected to account for 40% of the global CAD burden by 2020 [5]. More than 1.2 million South Asians live in Canada and are the fastest growing group of non-white Canadians [6]. Our previous study has shown that, compared to white Caucasians in Canada, South Asians suffer a 2.5 times excess prevalence of elevated glucose (dysglycemia), and CAD [7], and develop cardiometabolic risk factors (ie, abnormal glucose and lipids) at significantly lower body mass index (BMI) values [8].

Despite several previous studies showing excess cardiometabolic risk [4,9,10] and increased premature MI among South Asians [11], there is no routine screening process of South Asians for CAD despite the Canadian Cardiovascular Society recommendations to screen “high-risk” groups including South Asians [12]. Therefore, there is a need for routine screening of CAD risk factors in South Asian adults and to develop and test interventions to improve risk factors among South Asians. This is critical because collectively common risk factors (abnormal lipids, elevated glucose, elevated blood pressure, and abdominal obesity) account for over two-thirds of the population attributable risk of MI [4].

Several studies have shown that multimedia interventions to manage risk factors of common disorders and to modify health behaviors are effective [13-19]. Multimedia interventions include use of email messaging, text messaging, video- or computer-based education, and electronic personalized health records, which are attractive because they involve components of goal setting and feedback—key components of health behavior modification, are relatively cost efficient, and have the potential to be scalable to large numbers of individuals [20-25].

The use of MI risk tools to guide risk factor modification in cardiovascular prevention is increasing [26]. More recently the addition of genetic information into these risk tools has been evaluated. This has been made possible by the recent large-scale genetic studies that have identified common genetic variants associated with MI risk. The most robust genetic variant associated with increased risk for MI is a common polymorphism located on the short arm of chromosome 9 (9p21) [27,28]. This genetic variant is common in the general population, with 50% of people carrying one copy of the risk allele, which increases MI by 15-20%, and 25% of the population carrying two copies of the risk allele, which increases MI risk by 30-40% [29]. Further there is evidence to suggest that the MI risk associated with 9p21 may be modified by healthy dietary patterns [30]. While some recent studies have evaluated whether knowledge of genetic risk of a condition influences individuals’ behavior change [31,32], the results remain inconclusive. To our knowledge there have been no multimedia health behavior modification interventions, which have incorporated genetic risk information among South Asians at risk for MI.

### Objective

To address this gap we conducted a pilot study, the South Asian HeArt Risk Assessment (SAHARA) among at-risk South Asians living in Canada, to determine: (1) the feasibility of a culturally tailored multimedia intervention to induce positive behavioral changes associated with reduced MI risk factors, and (2) the effectiveness and acceptability of information communicated by individualized MI and genetic risk score (GRS) reports. Information generated from the SAHARA pilot study will directly inform the design of another randomized trial designed to test the effectiveness of this intervention to reduce MI risk among South Asians.

## Methods

### Study Design and Recruitment

The study is a randomized controlled pilot trial that was approved by the McMaster/Hamilton Health Sciences Research Ethics Board on June 3, 2009 (09-225).

Individuals were recruited from places of worship and community centers in Southwestern Ontario, Canada, during the period from January 16, 2011 to January 29, 2012. Recruitment clinics were setup in these “high-yield” locations at high yield times (following weekly ceremonies and scheduled activities) to maximize enrollment. The study team contacted community leaders in the recruitment locations to obtain permission to inform the congregation about the study, and this was done 1-2 weeks prior to the screening event.

### Eligibility

Men and women ≥30 years of age of South Asian ancestry, defined as people whose ancestors originate from the Indian subcontinent (India, Pakistan, Bangladesh, and Sri Lanka), were eligible for inclusion in the SAHARA pilot study. All participants were required to have access to email, cell phone with text messaging capability, or a smart phone (ie, a handheld device capable of sending and receiving text messages and searching the Internet such as an iPhone or Blackberry).

### Exclusion Criteria

Individuals who had suffered a previous MI, had coronary artery bypass graft (CABG) surgery, coronary angioplasty, or stroke, who were not permanent residents of Ontario, and who did not have an Ontario health card were excluded.

### Consent and Baseline Data Collection

Written informed consent, including consent to use of the health card number to facilitate future record linkage with health services databases, and to analyze DNA for genetic variants, was obtained from each participant. Information on risk factors including cholesterol status, diabetes, hypertension, current, former, and second-hand exposure to tobacco smoke, diet, physical activity, sedentary behaviors, and psychosocial stress questions was collected. Stages of change information based on Prochaska’s model of change [33] were also obtained for diet, physical activity, sedentary behavior, and smoking. Blood pressure (two measures 3 minutes apart using an automated OMRON device), body weight and height (to calculate BMI), waist and hip circumference, and body fat percentage using a digital bioelectrical impedance scale were measured. A 30-mL nonfasting blood sample was collected from all participants, and was processed onsite within 2 hours of collection. The blood samples were analyzed for apolipoprotein A1 and B, HbA1C, and the 9p21 single nucleotide polymorphism (SNP) (rs1333049) genotype using Taqman. All genotypes were in Hardy-Weinberg equilibrium (HWE) for the total sample (*P*>.05). Previous studies have reported a minimal difference in apolipoproteins’ levels when comparing fasting to nonfasting levels [34]. The remaining serum and plasma aliquots were placed in long-term storage for future study-related analysis.

### Risk Profile

**Figure 1:**
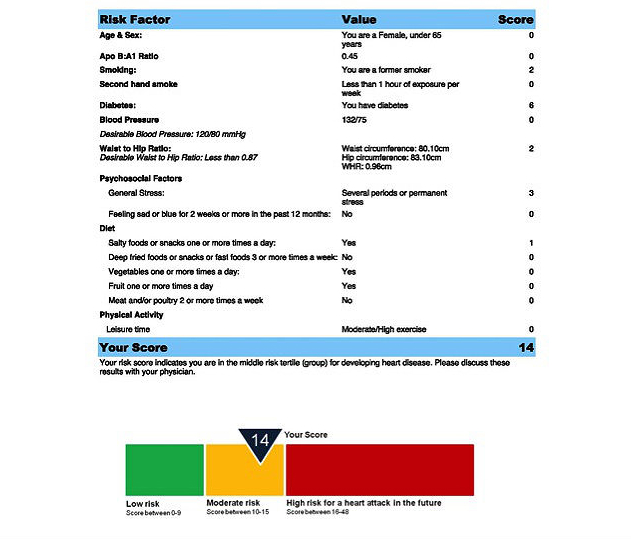
IHRS risk report example.

**Figure 2:**
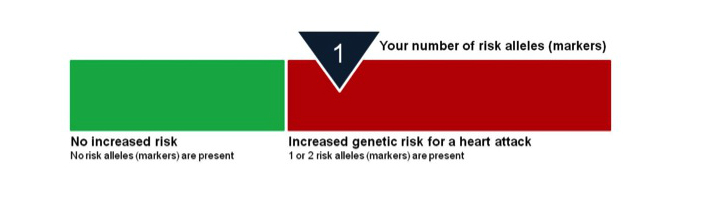
Genetic risk score. Through your blood work, we looked for a specific SNP in your DNA which has been shown to be a marker for heart attack risk. This SNP is located on chromosome 9, and is known as 9p21. The SNP is not within a gene itself, but is likely closely related to a gene which causes coronary artery disease. The 9p21 SNP has been shown to increase heart attack risk in several different ethnic groups, including South Asians. Based on your blood work, we determined if you did not have this SNP, only had it on one chromosome (inherited from one parent), or had it on two chromosomes (inherited from both parents). Having either one or two copies of this marker increases your genetic risk of having a heart attack.

Using the information collected at the baseline visit, a MI risk report was generated for each participant using the INTERHEART risk score (IHRS; Multimedia Appendix 1, which is a simple and valid risk factor scoring system developed and validated from the INTERHEART case-control study to assess MI risk in adult men and women [35]. This risk model included the following factors: apolipoprotein B-to-A1 ratio, smoking, second-hand smoke exposure, hypertension, HbA1c, abdominal obesity, physical inactivity, diet, and psychosocial factors. As part of the SAHARA pilot study the risk score report was pretested and modified in an easy to understand format that classifies individuals as low (0-9), moderate (10-15), or high (16-48) risk using a color visual display (Figure 1) (also see website [36]). In addition to IHRS, a GRS based on 9p21 genotype information was generated. The GRS was developed, pretested, and modified in an easy to understand format and classifies individuals who have 0, 1, or 2 risk alleles using a color visual display (Figure 2). The contents of the report were pretested in 2 focus groups conducted at a South Asian temple, and modified to the grade 5 reading level.

### Randomization

#### MyOSCAR-SAHARA

**Figure 3:**
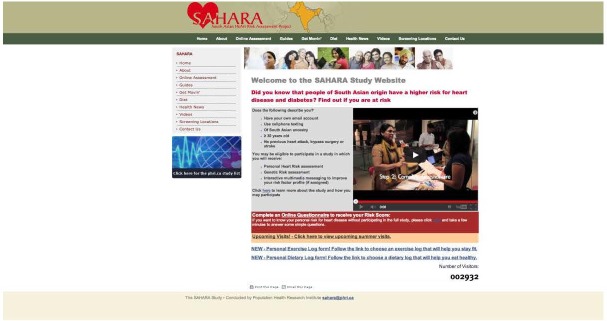
SAHARA home page.

**Figure 4:**
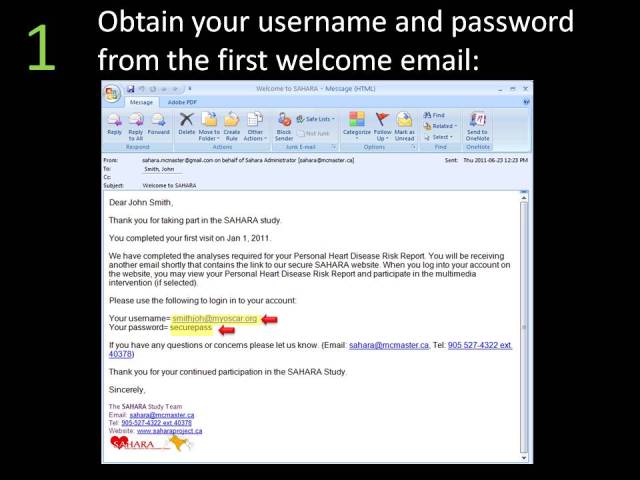
Welcome email.

**Figure 5:**
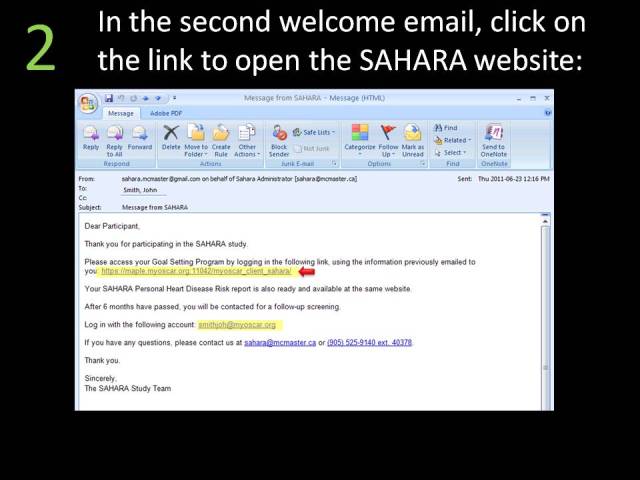
Second welcome email.

**Figure 6:**
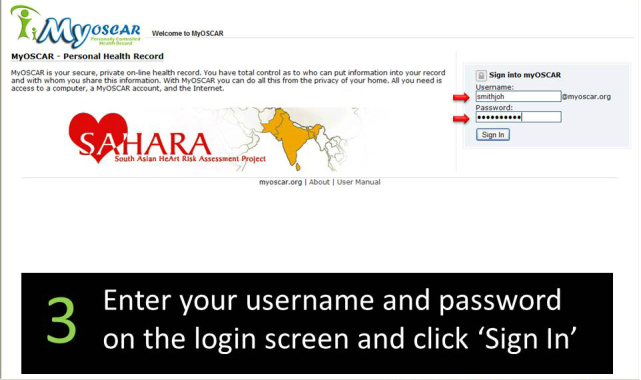
MyOSCAR Web login.

**Figure 7:**
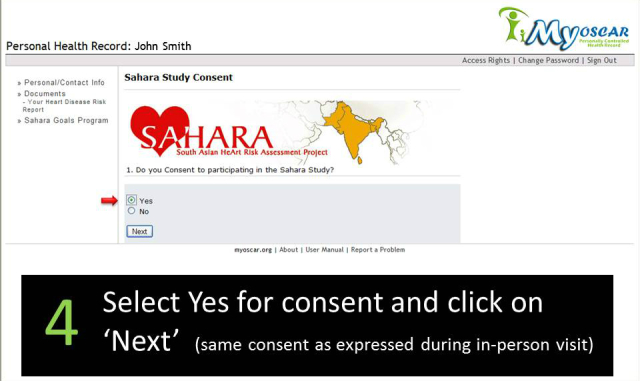
SAHARA Web consent and personal health record page.

Approximately 4-6 weeks after the screening visit was completed, participants were sent an email asking them to log onto the secured MyOSCAR-SAHARA website [37], to access their risk score results. If they were eligible for randomization (based on study inclusion criteria), they were prompted to click on a button that took them to a Web portal to be randomized to intervention or control (usual advice) groups using a computer-generated algorithm in OSCAR (Open Source Clinical Applications and Resources)—an open source software project launched by the Department of Family Medicine at McMaster University in Hamilton, ON, Canada, in 2002, designed for the delivery of evidence-based resources and decision support at the point of care for both patients and providers [37]. We used a specially constructed MyOSCAR-SAHARA personalized website to enable study participants’ to retrieve their results, and to set goals which triggered a series of goal-tailored health messages they received by email or text message (for screenshots, see Figures 3-7).

Intervention and control groups received the same baseline assessment and usual care while only the intervention group received the study intervention.

#### Intervention

Participants randomized to the intervention were prompted to choose a health goal on the website in the areas of (1) healthy diet, (2) physical activity, (3) reducing sedentary behaviors, and (4) smoking cessation, and were prompted to update their goals weekly on the website. Participants then received health messages via email or text, tailored to their chosen health goal on a daily basis. The messages were based on self-efficacy and social support concepts [38-41] to motivate subjects to make health behavior changes including providing advice and support regarding reduction of energy-dense, nutrient-poor foods (ie, fried, fast foods, sugary beverages, and desserts), advocating increased consumption of fruits and vegetables, encouraging sedentary individuals to minimize sedentary behaviors and increase regular physical activity, and encouraging smokers to quit smoking. Participants were given a choice of methods to receive the health messages by: (1) email sent to an account using a personal computer or a handheld device (eg, BlackBerry, iPhone, or other smartphones), or (2) text message (short message service, SMS) to a handheld device (any cell phone).

In addition to the health messages, a weekly health tip was sent to all intervention participants by email outlining a particular health topic related to healthy lifestyle or an analysis of a recent medical study reported in the press. All of these health messages were then posted on our public website (Figure 3). The components of the intervention are listed in Textbox 1

#### Control

Participants randomized to the control group were provided with advice on how to interpret their risk report, and if any significantly abnormal results were identified, they were encouraged to discuss them with their family doctor. All participants had access to the SAHARA website that contained health information regarding cardiovascular risk factors from a South Asian perspective [42]. This website includes information on culturally relevant healthy dietary habits, and the health benefits of regular physical activity. The site also includes a frequently asked questions section, and a mechanism for participants to ask our study team study-related questions and receive feedback.

**Multimedia intervention components of the SAHARA pilot study.**

Components for intervention participants:

- MyOSCAR-SAHARA Goal selection program: a tool which permits participants to select biweekly goals related to improving diet, increasing activity, decreasing smoking, and reducing sedentary behaviors
- Daily health messages—sent via email or text: messages provided tips on how to counter unhealthy habits and maintain healthy ones
- Biweekly reminders to pick a health goal and monitor progress on the goal
- Access to latest health information through personal MyOSCAR-SAHARA account
- Access to healthy living videos, such as yoga and other exercise regimens

### Website Usage and Adherence to Intervention “Fidelity”

Participants usage of the goal setting website was monitored centrally, and for those participants who did not log on to access their risk score reports or for intervention participants who had not set goals 2 weeks from the time they were prompted by email, a study team member attempted to reach them by telephone to encourage them to access their results and set goals. After 4 weeks if results had not been accessed from the website, a printed MI and genetic risk score report was mailed to participants’ home.

### Pilot Study Outcome Measures

The two outcome measures of the pilot study included: First, feasibility of the intervention, defined by: (1) success at transmitting risk score information and health messages via electronic media (website, email, and cell phone), (2) success at participants returning to use the website and set health goals, as this reflects the uptake of the intervention and helps to assess the effect of intervention on health behaviors, and (3) trend in the risk score change to indicate if the intervention leads to progressive health behavior change. Second, effectiveness and acceptability of risk score information was measured by: (1) participants’ knowledge of their risk score over time, (2) if this information induced positive behavior change, and (3) participants’ satisfaction with the information received.

### Follow-Up

All participants were followed up for a minimum of 6 months after randomization and repeat risk factor assessment was collected at the end of the study. End of study data were collected via face-to-face reassessment at the recruitment sites (238/324, 73.5%) and by telephone or mail (86/324, 26.5%). Repeat HbA1C and apolipoproteins A1 and B were also collected from participants who attended the face-to-face reassessment visit.

The reporting of this study follows the CONSORT-EHEALTH [43] guidelines Multimedia Appendix 2.

## Results

### Summary

**Figure 8:**
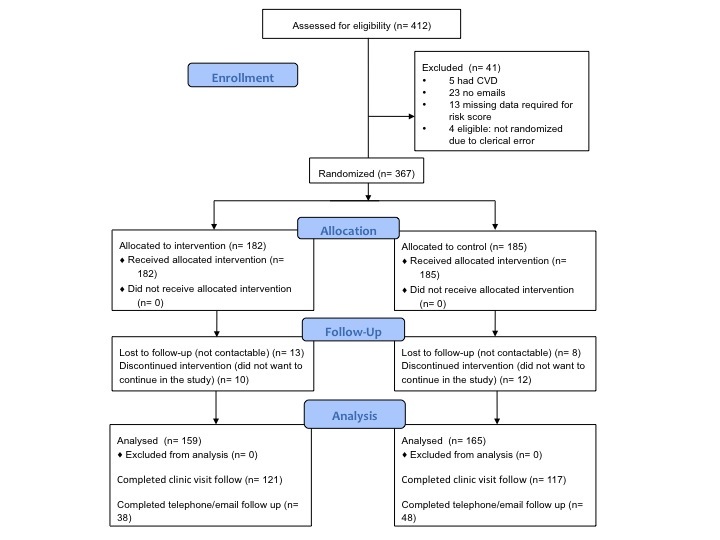
Participants' flow diagram.

Participants (n=412) were screened from 11 centers between January 2011 and January 2012. Among them, 41 were ineligible (5 had cardiovascular disease—CVD, 23 had no email accounts, 13 were missing information required for the risk score, and 4 were eligible but not randomized due to a clerical error), leaving 367 participants randomized into the pilot study. Follow-up data collection occurred between October 28, 2011 and November 11, 2012. The median time of follow-up is 280 days with the interquartile range (IQR) of 252-319 days follow-up. As shown in Figure 8, there were 43 participants (21/167, 12.6% and 22/204, 10.8% of the intervention and control group, respectively) who did not complete the follow-up (21 were not contactable and 22 participants withdrew from the study).

### Demographic and Social Characteristics

Participants’ characteristics are shown in Table 1. Briefly the mean age is 53.8 years (minimum age=30.0 years, maximum age=82.0 years, and median age=53.0 years), approximately half are women, and the majority of participants are immigrants to Canada. More than half reported speaking English at home, 88.7% (323/364) received more than secondary school education and 69.2% (254/367) are actively employed. More than 52.0% (191/367) are vegetarian; few (4/367, 1.1%) use or are exposed to tobacco, and approximately one-quarter (27.5%, 101/367) engage in regular physical activity. Further, more than 32.0% (117/367) are exposed to more than 2 hours of screen time per day. The mean BMI at baseline is 26.4 (SD 3.5) for men and 26.5 (SD 4.1) for women. Three quarters of participants have one or two risk alleles for the 9p21 genetic variant.

### Risk Factor Information at Baseline and End of Study

Table 2 shows participants’ risk factors at baseline and follow-up. Over a quarter of all participants had hypertension and elevated cholesterol at baseline, and 13.8% (44/319) reported having type 2 diabetes. Over two thirds of participants are inactive at leisure time, the mean servings of fruits and vegetables consumed daily are 2 and 3, respectively, and more than 27.1% (86/317) of participants reported having stress and depressive symptoms. Objective study measures including HbA1c (mean 5.9, SD 0.8; apolipoprotein B-to-A1 ratio: mean 0.68, SD 0.18) and waist-to-hip ratio (WHR, men: mean 0.95, SD 0.05; women: mean 0.88, SD 0.07) indicate that the cohort has a moderate risk for MI, with the mean IHRS being 13.0 (SD 5.8).

### Feasibility: Success at Transmitting Messages Via Electronic Media

All participants were required to have an email access (including shared family email if they choose to use this email account) to be eligible for this study. The majority of participants (352/367, 96%) had personal email access (Table 3). Most participants had no difficulty logging into website or viewing their results, although 23% (74/324) reported having some technical problems with the website, which inhibited the risk score report and health messages delivery.

### Success at Participants Returning to Use the Website and Set Health Goals

**Figure 9:**
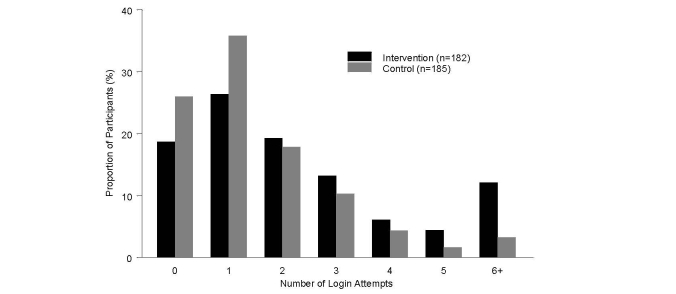
Summary of login attempts to website over the course of the pilot study.

Participants use of the MyOSCAR-SAHARA website was monitored to determine how many participants logged on to the website to view their risk score reports and in the case of intervention participants, to set health goals. The login to the study MyOSCAR-SAHARA website was low for both groups (82/182, 45.1% of intervention and 115/185, 62.7% of control groups did not logon or used the website only once). The mean number of login attempts of the intervention group mean was 2.64 (SD 3.17, median 2.0) and the control group was 1.63 (SD 2.14, median 1.0). The difference between intervention and control groups login was statistically significant as expected since the intervention group was asked to set goals (Wilcoxon two-sample test, *P*=.0003). Figure 9 shows the frequency of login by intervention and control groups. On average the intervention group selected 1.12 goals (SD 1.67, median 1.0).

**Table 1:** Demographic characteristics.

Characteristics		Overall	Intervention	Control	
Number of participants		367	182	185	
Age in years, mean (SD)		53.8 (11.4)	54.6 (11.5)	53.0 (11.3)	
Median age (min, max)		53.0 (30.0, 82.0)	55.0 (31.0, 81.0)	53.0 (30.0, 82.0)	
Male/female (%)		176 (48.0)/191 (52)	84 (46.2)/98 (53.8)	92 (49.7)/93 (50.3)	
**Ancestral country of origin (%)**				
	India	327 (89.1)	163 (89.6)	164 (88.6)
	Pakistan	4 (1.1)	2 (1.1)	2 (1.1)
	Sri Lanka	4 (1.1)	2 (1.1)	2 (1.1)
	Other	32 (8.7)	15 (8.2)	17 (9.2)
Place of birth—Canada, (%)		9 (2.5)	1 (0.6)	8 (4.4)	
Language spoken at home—English, (%)		188 (52.2)	93 (52.0)	95 (52.5)	
Married (%)		337 (92.1)	167 (91.8)	170 (92.4)	
Post-secondary education (%)		323 (88.7)	156 (86.7)	167 (90.8)	
Employed (%)		254 (69.2)	119 (65.4)	135 (73.0)	
Household income >CDN$ 60,000/year (%)		218 (61.6)	106 (59.6)	112 (63.6)	
Alcohol consumption ≥1 drink per day (%)		23 (6.4)	11 (6.0)	12 (6.8)	
Vegetarian (%)		191 (52.5)	90 (49.5)	101 (55.5)	
Daily activity mild/none (%)		266 (72.5)	125 (69.8)	141 (77.0)	
Screen time mean minutes/day (SD)		140.1 (130.7)	133.8 (121.5)	146.3 (139.2)	
BMI—male, mean (SD)		26.4 (3.5)	26.5 (3.6)	26.4 (3.3)	
BMI—female, mean (SD)		26.5 (4.1)	26.2 (3.7)	26.8 (4.5)	
One or two risk alleles of 9p21 (%)		261 (73.3)	130 (74.3)	131 (72.4)	

**Table 2:** Baseline and follow-up risk factors in intervention and control groups.

Risk factor	Intervention			Control			
	Baseline	Follow-up	*P* value^a^		Baseline	Follow-up	*P* value^a^
Apolipoprotein B/apolipoprotein A1 ratio (SD)	0.66 (0.19)	0.67 (0.18)	.42		0.70 (0.18)	0.71 (0.20)	.59
HbA1c (SD)	5.9 (0.8)	5.9 (0.8)	.81		5.8 (0.8)	5.9 (0.9)	.01
Self-reported diabetes,^b^ n^c^ (%)	31 (20.0)	36 (23.2)	.03		13 (7.9)	16 (9.7)	.08
Self-reported hypertension, n^c^ (%)	43 (28.1)	46 (30.1)	.08		36 (22.4)	42 (26.1)	.01
Elevated BP,^d^ n (%)	35 (30.4)	25 (21.7)	.06		31 (27.0)	22 (19.1)	.08
Mean SBP (SD) mm Hg	128 (18)	124 (16)	.008		127 (19)	123 (17)	.003
Mean DBP (SD) mm Hg	81 (10)	79 (10)	.006		82 (11)	79 (11)	<.0001
Waist-to-hip ratio—male, mean (SD)	0.95 (0.06)	0.96 (0.06)	.68		0.95 (0.05)	0.94 (0.06)	.07
Waist-to-hip ratio—female, mean (SD)	0.89 (0.07)	0.88 (0.07)	.12		0.87 (0.06)	0.87 (0.06)	.62
Stress in last year at baseline and in last 6 months at follow-up, n (%)	48 (30.8)	29 (18.6)	.001		52 (32.1)	33 (20.4)	.002
Depression for ≥2 weeks in last year at baseline and last 6 months at follow-up, n (%)	50 (32.3)	24 (15.5)	.0001		36 (22.2)	16 (9.9)	.0009
Mean servings of fruits/day (SD)	2.0 (1.2)	2.0 (1.2)	.90		2.0 (1.2)	1.9 (1.4)	.67
Mean servings of vegetables/day (SD)	3.0 (1.7)	2.8 (1.8)	.24		3.0 (1.9)	2.9 (1.9)	.83
Mean servings of deep fried foods/snacks per day (SD)	0.3 (0.5)	0.2 (0.2)	.0005		0.3 (0.4)	0.2 (0.4)	.003
Moderate/very active in leisure time, n (%)	58 (37.9)	88 (57.5)	<.0001		47 (29.0)	77 (47.5)	<.0001
Self-reported high cholesterol,^c^ n (%)	42 (28.2)	49 (32.9)	.008		37 (22.6)	44 (26.8)	.008
IHRS^e,f^ (SD)	13.4 (5.8)	12.0 (5.8)	.002		12.6 (5.9)	11.7 (5.9)	.05

^a^Pairwise comparison of data using paired *t*-test for continuous measures and McNemar’s test for categorical measures.

^b^Prevalence of events at follow-up includes baseline plus additional new events since baseline. Therefore the prevalence of diabetes, hypertension, and high cholesterol are higher at follow-up.

^c^Number of participants with data available for the specific variable.

^d^Bloodpressure (BP) was measured at baseline and follow-up. Elevated BP is >140/90.

^e^IHRS: INTERHEART risk score.

^f^No significant difference in change between the intervention and control group (*P*=.70).

**Table 3:** Electronic access and reported technical difficulties.

Baseline	Overall, n=367 n (%)	Intervention, n=182 n (%)	Control, n=185 n (%)
Personal email access	353 (96.2)	176 (96.7)	177 (95.7)
Smart phone access	73 (19.9)	41 (22.5)	32 (17.3)
Cell phone access	177 (48.2)	98 (53.8)	79 (42.7)
Receive and send text messages	127 (34.6)	141 (38.5)	57 (30.8)
Check email multiple times per day	148 (40.3)	79 (43.4)	69 (37.3)
Participants who completed follow-up	324 (88.3)	159 (87.4)	165 (89.2)
Problems accessing results on website	25 (7.7)	9 (5.7)	16 (9.7)
Logon difficulties to website	37 (11.4)	17 (10.7)	20 (12.1)
Did not receive email with instruction on logon	11 (3.4)	4 (2.5)	7 (4.2)
Instructions were unclear	10 (3.1)	4 (2.5)	6 (3.6)
MyOSCAR-SAHARA website was difficult to use	13 (4.0)	4 (2.5)	9 (5.5)
Total problems with MyOSCAR-SAHARA website	74 (22.8)	31 (19.5)	43 (26.1)

### Signal That Intervention Leads to Behavioral Changes

Both the intervention and control group showed a reduction at follow-up in blood pressure, and reported less stress and depression compared to baseline. There was also an improvement in physical activity and reduction in fried food and snacks consumption in both groups at follow-up. Comparing follow-up to baseline score change, the intervention group had a significant reduction in their IHRS score at follow-up (intervention group baseline IHRS: mean 13.4, SD 5.8; follow-up IHRS: mean 12.1, SD 5.9, *P*=.002), and a trend was seen in the control group (baseline IHRS: mean 12.6, SD 5.8; follow-up IHRS: mean 11.7, SD 5.9, *P*=.05) (see Table 2), though these results were not statistically significant.

### Risk Score

#### Risk Report Feedback

Participants were asked to acquire their risk score reports (IHRS and GRS) following the baseline assessment by logging onto the MyOSCAR-SAHARA website. If they did not retrieve it, it was mailed to their homes. At the end of the study, participants were asked about their knowledge and recall of their risk scores (Tables 4 and 5). Overall while participants reported appreciating receiving their risk information, the recall between baseline and end of study of risk status was low. For example, of 68 participants who were told they were high-risk at baseline, only 11 recalled this correctly (11/68, 16.2%), 17 recalled being moderate risk (17/68, 25.0%), 3 recalled it being low risk (4/68, 4.4%), and 37 could not recall their risk score (37/38, 54.4%) at all. Similarly only 7.3% (5/68) of participants recalled their increased genetic risk score accurately at follow-up (Table 5).

#### Risk Scores and Motivation to Change

**Figure 10:**
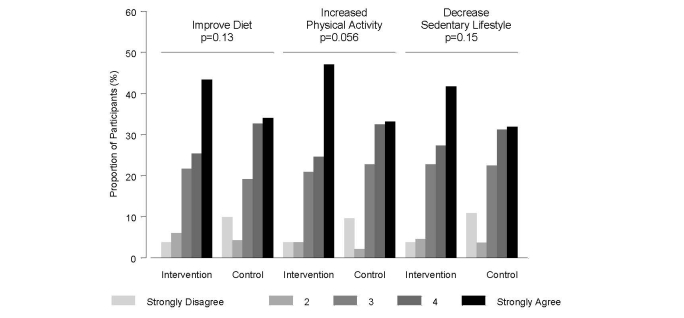
Motivation to change behavior based on risk score reports.

Figure 10 shows individuals reporting that knowledge of their risk for MI could be a motivator to change health behaviors including diet and physical activity. There was a trend (*P*=.06) showing the intervention group as compared to the control group was more likely to agree that the risk score was a motivator for increasing health behaviors especially for physical activity, though these results were not statistically significant (Figure 10).

### Stages of Change

We assessed the stages of change for three main domains: diet, physical activity, and weight loss. Although we also included smoking, only three individuals are current smokers in this sample. Overall, more than 13.9% (51/367) of participants progressed from inactive (precontemplation, contemplation, and preparation) to the active (action, maintenance) stage in diet and physical activity, and 12.5% (46/367) progressed to the active stage in weight loss plans; however, no significant differences were observed between intervention and control groups (Table 6).

### Exit Survey

At the end of the pilot study, feedback from the study participants was obtained by asking all participants about their experiences of participating in the pilot study. The main feedback included: (1) daily messages were too frequent which could potentially lead them to ignore the messages; (2) phone calls to remind participants to login to the study website were too frequent, while others reported that there was not enough in-person contact and would have liked to have a mid-program visit that with more face-to-face contact with the study team; and (3) IHRS and GRS reports should be sent via email and remove the website login component. Most of the participants reported that participation in the SAHARA study was worthwhile for them. Table 7 shows summary of the exit survey.

**Table 4:** Agreement between MI risk score results and participants recall of risk score at follow-up.

Actual IHRS score category at baseline	Recall of risk score category at follow-up				
	Low	Moderate	High	Do not know	Total
Low	47	15	2	63	127
Moderate	11	46	6	59	122
High	3	17	11	37	68
Total	61	78	19	160	318

**Table 5:** Agreement between genetic risk score results and participants recall of risk score at follow-up.

Actual GRS score category at baseline	Genetic risk category recall at follow-up			
	Not increased	Increased	Do not know	Total
Not increased (0 risk alleles)	22	6	56	84
Increase (1 or 2 risk alleles)	38	16	172	226
Total	60	22	228	310

**Table 6:** Stages of change^a^: moving from inactive to active stage by intervention at follow-up.

Domain	Overall, %	Intervention,%	Control, %	*P* value^b^
Diet	14.9	15.7	14.1	0.69
Weight loss	12.5	13.7	11.4	0.53
Physical activity	14.2	15.5	12.9	0.51

^a^Stages of change levels: 1=precontemplation, 2=contemplation, 3=preparation, 4=action, 5=maintenance. Inactive=levels 1-3, active=levels 4-5.

^b^These results were obtained from chi-square tests.

**Table 7:** Exit survey (n=317).

Rank		Intervention, n (%)	Control, n (%)
**(A) Did you find participation in SAHARA to be worthwhile?** ^a^			
	Number of participants	155 (48.8)	162 (51.1)
	Very worthwhile	75 (48.4)	78 (48.2)
	4	47 (30.3)	45 (27.8)
	3	24 (15.5)	19 (11.7)
	2	8 (5.2)	10 (6.2)
	Not at all worthwhile	1 (0.6)	10 (6.2)
**(B) Did you succeed in setting and achieving your health goals?** ^b^			
	Number of participants	153 (48.3)	157 (49.5)
	Very successful	27 (17.7)	27 (17.2)
	4	60 (39.2)	50 (31.9)
	3	51 (33.3)	38 (24.2)
	2	9 (5.9)	25 (15.9)
	Not at all successful	6 (3.9)	17 (10.8)

^a^There was no significant statistical difference between intervention and control groups in their view of study participation (*P*=.09; obtained from chi-square tests).

^b^The intervention group reported that they were more likely to be successful in achieving their goals than the control group (*P*=.004; obtained from chi-square tests).

## Discussion

### Principal Findings

We observed that a multimedia health behavior intervention is feasible in a South Asian population at risk for MI. While participants reported being motivated by receiving the risk score information, a number of features of the SAHARA intervention require optimization prior to assessing its effectiveness in MI risk factor reduction.

Most participants had access to email, Internet, and text messages and had no difficulty receiving email or text messages. However, our requirement of participants to proactively logon to the website to receive their risk reports, and to set goals was problematic with 23.9% (88/367) of the study participants reporting technical difficulties. It is likely that this contributed to the low number of goals chosen over the course of the follow-up, and reduced the interventions potential impact on changing health behaviors. In addition, participants received the study messages either by email/text checked on a mobile device or emails checked on a fixed device. These different methods of receiving messages may have also impacted the uptake of the study intervention. The anticipated difference would be based on the fact that the mobile device message would likely be received in real time or close to it, whereas the fixed device message might not be received immediately, although it may reach people when they’re more ready to act on the information (ie, they have specifically chosen to sit down at the computer, as compared to a mobile device when the email/text may arrive when the person is doing something else). In this study it is not known the impact of receiving messages via mobile or a fixed device on the intervention uptake and outcome. Despite these technical challenges, the intervention group showed a significant reduction in the IHRS score at follow-up, and were more likely than control subjects to report that their personalized risk scores motivated them to increase their healthy dietary choices, physical activity, and reduce sedentary behaviors. The greater engagement of the intervention group in the study, their receipt of regular messages and reminders to change their health behaviors, may explain this difference.

Based on the participants’ feedback from the exit survey, the use of a website health behavior intervention, which requires participants to logon to a website, reduces the chances that participants will be engaged in the study. In our study, 54.9% (100/182) of the intervention group logged onto the website at least twice, which is in keeping with previous studies using Internet-based intervention to aid smoking cessation [44]. A systematic review and meta-analysis of Web-based intervention studies to induce behavioral changes reported that the average logon to website/person/study duration in weeks varies from 2.6 logons/person/32 weeks in a study of depression to 1008 logons/person/36 weeks in a study of HIV. In addition the average time spent on website in minutes per person varied from 4.5 to 45 minutes/person [45]. Furthermore, even when information is sent directly to participants by email, the rate of opening the email is variable. For example in a study of 345 men and women where daily email messages were sent to improve employees’ diet and physical activity behaviors in the workplace, only 68.9% (238/345) of the emails were opened by study participants [23], even though all study participants worked in the same office and had a computer at their desk. This is consistent with other studies using Web-based interventions where an uptake of only 62% was reported [46]. To optimize the uptake of the intervention for the main SAHARA trial we will ask participants to set their goals at the baseline interview, we will remove the logon to website requirement to access risk score reports, and we will deliver the reports directly to participants by emails. These components will be followed by telephone calls and one face-to-face visit mid-way through the study, to ensure receipt and knowledge of risk scores, and to maintain participant interest in the study.

Individuals who participated in this pilot study were at moderate risk of MI based on their baseline IHRS compared to risk score values reported in the validation study [35]. Both the intervention and control groups made some progress in changing their health behaviors and in general participants reported the information they received was useful. Despite participants claiming that their risk reports motivated behavioral changes, half of the participants could not recall their risk report at 6 months. The poor risk score recall may reflect low health literacy (ie, the degree to which individuals can obtain, process, and understand the basic health information) and numeracy (how individuals interpret medical risk information) [47]. However, our sample was of high socioeconomic status, well educated, and we pretested our risk score information in focus groups and presented the information (Figure 2) at the grade 5 reading level. Thus, we attempted to minimize low health literacy and numeracy as possible barriers to understanding risk score information. It is also possible that the active phenomenon of resistance to retain negative information about one’s health to maintain an optimistic view of future health was at play. Such views have been described as psychological defense mechanisms [48]; however, it is difficult to confirm if such views hold in the current study. In addition, the low risk score recall may also represent the phenomenon of “unrealistic optimism” whereby individuals display an optimism bias when evaluating own susceptibility to risk [49]. However, this view does not explain the poor recall of low-risk reports.

The low recall rate of health information received, including in face-to-face counseling, is not uncommon. In a large study investigating the recall of health advice given face-to-face to patients (n=3261) who participated in the EuroHeart Failure Survey 12 weeks following discharge, only 57.8% (1885/3261) of patients recalled advice on exercise, 54.9% (1793/3261) recalled advice on diet, 41.9% (1369/3261) recalled advice on smoking, and only 38.9% (1271/3261) recalled advice on alcohol [50]. Nonetheless in our study, participants reported that knowledge of their risk factor and genetic risk score were motivators to improve their health behaviors even if they could not recall their exact risk category. It is possible that recall may vary by the type of information provided to participants, and recall may decrease over time. For example, patients with type 2 diabetes are more likely to recall health recommendations regarding medications than regarding health behaviors [51], and provision of genetic risk information to smokers regarding their risk of lung cancer showed early high recall of risk status yet lower recall with increasing duration of follow-up [52]. We hypothesized that genetic information may motivate behavior change differently than nongenetic health information because of the highly personalized nature of the information [47]. In a recent study among patients with type 2 diabetes who participated in a lifestyle modification trial in which genetic information was provided in a gene score, almost all participants (98%) reported that high-risk genetic results would increase their motivation for lifestyle modification. On the other hand their response to receiving low-risk genetic results varied, with some reporting that low-risk genetic status would decrease their motivation to take on lifestyle changes. However, their reported response has not yet been correlated to their actual change in risk factors as this study is on-going [53]. Future studies, including the main SAHARA trial must assess if provision of genetic risk information is strongly correlated with changes in risk factors and clinical outcomes.

We assessed the stage of change transition over the course of the follow-up. It is known now that the stages of change are not linear and individuals do not progress from one stage to the next as originally proposed [33]. Rather, these stages follow a spiral model with relapses that resets the process back to the precontemplation stage [54]. Despite these challenges, the stages of change model is widely used and accepted as a useful measure to assess motivation to change and the impact of this motivation on achieving the desired behavioral modification [55]. We observed that 25.8% (94/367) of the participants progressed forward in stages of change relating to physical activity, while 18.2% (67/367) regressed in their stages of change. Overall however more than 12.5% (46/367) progressed from the inactive to an active stage at the end of study in all three domains (physical activity, diet, and weight loss). No difference between intervention and control subjects in stages of change transitions was observed.

### Limitations

Our pilot study had a number of strengths, which include recruitment of an apparently healthy population sample of reasonable size, and prospective measurement of health behaviors that included objective measurements (ie, lipid, blood pressure, and anthropometric measurements). Some limitations of our intervention occurred including the technical challenges of logging onto the website, the low rate of logons to set health goals, and relatively poor recall of personal risk at follow-up. Despite these, a significant reduction in the MI risk score was observed in follow-up. In addition the SAHARA study population may not be representative of all South Asians in Canada; however, the socioeconomic characteristics of SAHARA participants are similar to findings from previous health surveys in Ontario [56].

### Modifications to the SAHARA Trial Intervention

We have taken a number of steps to optimize the SAHARA intervention prior to testing its effectiveness in MI risk reduction in a future trial. These changes include: (1) risk reports and randomization status will be emailed directly to participants, (2) the number of health goals participants can focus on has been reduced from 4 to 2, with only one being chosen at one time for a 6-month duration, (3) the duration of follow-up will be extended to 12 months with baseline, 6 months and 12 months face-to-face visits occurring, (4) increasing the frequency of in-person contacts to improve adherence to the intervention and interest in the program, (5) health tips will be tailored to each participant based on the goal selected and their readiness to change, and (6) the frequency of messages will be reduced from daily to weekly and sent at a time of day chosen by participants.

### Conclusion

A multimedia intervention is feasible and has the potential to induce positive health behavior changes aimed at reducing MI risk. Information generated from the SAHARA pilot has directly informed the design of the main randomized trial designed to test the effectiveness of a multimedia behavioral intervention to reduce MI risk among South Asians.

## Multimedia Appendix 1

The INTERHEART Risk Score.

## Multimedia Appendix 2

CONSORT-EHEALTH checklist V1.6.2 [43].

## Abbreviations

BMI: body mass index
CABG: coronary artery bypass graft
CAD: coronary artery disease
GRS: genetic risk score
HWE: Hardy-Weinberg equilibrium
IHRS: INTERHEART risk score
IQR: interquartile range
MI: myocardial infarction MI
OSCAR: Open Source Clinical Applications and Resources
SAHARA: South Asian HeArt Risk Assessment
SMS: short message service
SNP: single nucleotide polymorphism
WHR: waist-to-hip ratio
