# Supplementary material for: South Asian Heart Risk Assessment (SAHARA): Randomized Controlled Trial Design and Pilot Study
Source: JMIR Res Protoc. 2013 Aug 20;2(2):e33. doi: 10.2196/resprot.2621 (PMC3757993; doi:10.2196/resprot.2621)
Supplement: Supplementary file 1 [file resprot_v2i2e33_app1.pdf]

Risk factor	*Question*		*Points for the answer*	**Points for each section**
**Age Group**	Are you a man 55 years or older OR woman 65 years or older?		2	**Points:**
	OR Are you a man younger than 55 years or woman younger than 65 years		0	
**Apolipoprotein B:A1 ratio**	Pick one only:	Q1: Less than 0.64	0	**Points:**
		Q2: 0.64 - 0.80	2	
		Q3: 0.80 - 0.99	3	
		Q4: greater than or =0.99	7	
**Smoking. Pick the description which matches you best:**	I never smoked		0	**Points:**
	OR I am a former smoker (last smoked more than 12 months ago)		2	
	OR I am a current smoker or I smoked regularly in the last 12 months, and I smoke…	1-5 cigarettes/d	2	
		6-10 cigarettes/d	4	
		11-15 cigarettes/d	6	
		16-20 cigarettes/d	7	
		More than 20 cigarettes/d	10	
**Second hand smoke**	Over the past 12 months, what has been your typical exposure to other people's tobacco smoke?	Less than 1 hour or exposure per week or no exposure	0	**Points:**
		OR One or more hours of secondhand smoke exposure per week	2	
**Glucose Status (HbA1c%)**	Pick one only:	Q1: Less than 5.5	0	**Points:**
		Q2: 5.5 – 5.8	1	
		Q3: 5.8 – 6.1	2	
		Q4: greater than or =6.1	6	
**Blood Pressure**	Do you have Hypertension	No	0	**Points:**
		SBP>140 or DBP>90	3	
		Hypertension on Medication	6	
**Waist to hip ratio**	Pick one only:	Q1: Less than 0.875	0	**Points:**
		Q2: 0.875 - 0.920	2	
		Q3: 0.920 - 0.967	3	

		Q4: greater than or =0.967	5	
**Stress**	How often have you felt stress in the last year? Pick one only	Never or some periods	0	**Points:**
		OR Several periods of stress or permanent stress	2	
**Depression**	During the past 12 months, was there ever a time when you felt sad, blue, or depressed for two weeks or more in a row?	No	0	**Points:**
		Yes	3	
**Dietary factors. Pick one answer for each food group**	Do you eat salty food or snacks one or more times a day	No	0	**Points:**
		Yes	1	
	Do you eat deep fried foods or snacks or fast foods 3 or more times a week?	No	0	**Points:**
		Yes	1	
	Do you eat fruit one or more times daily?	No	1	**Points:**
		Yes	0	
	Do you eat vegetables one or more times daily?	No	2	**Points:**
		Yes	0	
	Do you eat meat and/ or poultry 2 or more times daily?	No	0	**Points:**
		Yes	2	
**Physical activity**	How active are you during your leisure time?	I am mainly sedentary or perform mild exercise (requiring minimal effort)	2	**Points:**
		I perform moderate to high level exercise	0	

Table 1 The INTERHEART Risk Score

Legend: The categories of the risk factors are presented in the first column, and the specific questions to be asked in the middle columns. Only one answer need to be chosen for every question, and inserted into the “points” column. All questions must be answered for the most accurate risk score estimate.
