# Supplementary material for: South Asian Heart Risk Assessment (SAHARA): Randomized Controlled Trial Design and Pilot Study
Source: JMIR Res Protoc. 2013 Aug 20;2(2):e33. doi: 10.2196/resprot.2621 (PMC3757993; doi:10.2196/resprot.2621)
Supplement: Supplementary file 2 [file resprot_v2i2e33_app2.pdf]

CONSORT-EHEALTH Checklist V1.6.2 Report

(based on CONSORT-EHEALTH V1.6), available at [http://tinyurl.com/consort-ehealth-v1-6].

2621

Date completed

3/15/2013 23:30:49

by

Zainab Samaan

South Asian Heart Risk Assessment (SAHARA): Randomized Trial Design and Pilot Study Results

TITLE

1a-i) Identify the mode of delivery in the title

The title does not state the mode of delivery because the mode of delivery is mixed media.

1a-ii) Non-web-based components or important co-interventions in title

1a-iii) Primary condition or target group in the title

South Asian Heart Risk Assessment (SAHARA): Randomized Trial Design and Pilot Study Results

ABSTRACT

1b-i) Key features/functionalities/components of the intervention and comparator in the METHODS section of the ABSTRACT

Background: People of South Asian origin suffer a high burden of premature myocardial infarction (MI). Prior studies have shown that multimedia interventions are effective and feasible in inducing health behavior changes.

Objectives: Among at risk South Asians living in Canada, our objectives are to determine: 1) the feasibility of a culturally tailored multimedia intervention to induce positive behavioral changes associated with reduced MI risk factors, and 2) the effectiveness and acceptability of information communicated by individualized MI and genetic risk score reports.

1b-ii) Level of human involvement in the METHODS section of the ABSTRACT

Participants were randomly allocated to receive a multimedia intervention or control. The intervention group selected health goals and received personalized health messages to promote adherence to their selected goals. After six months all participants had their MI risk factors repeated.

1b-iii) Open vs. closed, web-based (self-assessment) vs. face-to-face assessments in the METHODS section of the ABSTRACT

Methods: The South Asian HeArt Risk Assessment (SAHARA) pilot study enrolled 367 individuals of South Asian origin recruited from places of worship and community centers in Ontario, Canada. MI risk factors including the 9p21 genetic variant status were provided to all participants after the baseline visit.

1b-iv) RESULTS section in abstract must contain use data

Results: The mean age of participants was 53.8 years (SD 11.4), 52% were women, and 97.5% were immigrants to Canada. The mean INTERHEART risk score was 13.0 (SD 5.8) and 73.3% had one or two copies of the risk allele for the 9p21 genetic variant. Both the intervention and control groups made some progress in health behavior changes related to diet and physical activity over six months. Participants reported that their risk score reports motivated behavioral changes, although half of the participants could not recall their risk scores at the end of study evaluation. Some components of the multimedia intervention were not widely used such as logging onto the website to set new health goals, and participants requested having more personal interactions with the study team.

1b-v) CONCLUSIONS/DISCUSSION in abstract for negative trials

Conclusions: Some, but not all, components of a multimedia intervention are feasible and have the potential to induce positive health behavior changes. MI and genetic risk score reports are desired by participants although their impact on inducing sustained health behavior change requires further evaluation. Information generated from this pilot study has directly informed the design of a larger randomized trial designed to reduce MI risk among South Asians.

INTRODUCTION

2a-i) Problem and the type of system/solution

Myocardial infarction (MI) due to coronary artery disease (CAD) remains a major cause of death globally.[1] The rising prevalence of overweight, obesity and type 2 diabetes is predicted to potentiate the CAD epidemic in developing countries.[2] South Asians, people who originate from the Indian subcontinent, suffer a high burden of premature MI,[3, 4] and are projected to account for 40% of the global CAD burden by 2020.[5] More than 1.2 million South Asians live in Canada and are the fastest growing group of non-white Canadians.[6]

Several studies have shown that multimedia interventions to manage risk factors of common disorders and to modify health behaviours are effective.[9-15] Multimedia interventions include use of email messaging, text messaging, video or computer based education and electronic personalized health records, which are attractive because they involve components of goal setting and feedback – key components of health behavior modification, are relatively cost efficient, and have the potential to be scalable to large numbers of individuals.[16-21]

2a-ii) Scientific background, rationale: What is known about the (type of) system

The use of MI risk tools to guide risk factor modification in cardiovascular prevention is increasing.[22] More recently the addition of genetic information into these risk tools has been evaluated. This has been made possible by the recent large-scale genetic studies that have identified common genetic variants associated with MI risk. The most robust genetic variant associated with increased risk for MI is a common polymorphism located on the short arm of chromosome 9 (9p21).[23, 24] This genetic variant is common in the general population, with 50% of people carrying one copy of the risk allele, which increases MI by 15-20%, and 25% of the population carrying two copies of the risk allele, which increases MI risk by 30-40%.[25] Further there is evidence to suggest that the MI risk associated with 9p21 may be modified by healthy dietary patterns.[26] While some recent studies have evaluated whether knowledge of genetic risk of a condition influences individuals' behavior change,[27, 28] the results remain inconclusive. To our knowledge there have been no multimedia health behavior modification interventions, which have incorporated genetic risk information among South Asians at risk for MI.

METHODS

3a) CONSORT: Description of trial design (such as parallel, factorial) including allocation ratio

To address this gap we conducted a pilot study, the South Asian HeArt Risk Assessment (SAHARA) among at risk South Asians living in Canada, to test: 1) The feasibility of a culturally tailored multimedia intervention to induce positive behavioral changes associated with reduced MI risk factors, and 2) The effectiveness and acceptability of information communicated by individualized MI and genetic risk score reports. Information generated from the SAHARA pilot will directly inform the design of a larger randomized trial designed to test the effectiveness of this intervention to reduce MI risk among South Asians.

3b) CONSORT: Important changes to methods after trial commencement (such as eligibility criteria), with reasons

No changes were made as this is the pilot phase of the trial. Changes to the main trial are proposed in the manuscript based on experience gained from pilot study.

3b-i) Bug fixes, Downtimes, Content Changes

Proposed changes are explained in the manuscript for the main trial:
Modifications to the SAHARA Large Trial Intervention: We have taken a number of steps to optimize the SAHARA intervention prior to testing its effective in MI risk reduction in a larger trial. These changes include: i. risk reports and randomization status will be emailed directly to participants, ii. the number of health goals participants can focus on has been reduced from 4 to 2, with only one being chosen at one time for a 6 month duration. iii. the duration of follow-up will be extended to 12 months with baseline, 6 months and 12 months face-to-face visits occurring, iv. increasing the frequency of in-person contacts to improve adherence to the intervention and interest in the program, v. health tips will be tailored to each participant based on the goal selected and their readiness to change, and vi. the frequency of messages will be reduced from daily to weekly and sent at a time of day chosen by participant.

4a) CONSORT: Eligibility criteria for participants

Eligibility
Men and women ≥30 years of age of South Asian ancestry, defined as people whose ancestors originate from the Indian subcontinent (India, Pakistan, Bangladesh and Sri Lanka) were eligible for inclusion in the SAHARA pilot study. All participants were required to have access to email, cell phone with text messaging capability, or a smart phone (i.e. a hand held device capable of sending and receiving text messages and searching the internet such as an iPhone or Blackberry).
Exclusion criteria
Individuals who had suffered a previous MI, had CABG surgery, coronary angioplasty or stroke, who were not permanent residents of Ontario, and who did not have an Ontario health card were excluded.

4a-i) Computer / Internet literacy

Website usage and adherence to intervention “fidelity”
Participants usage of the goal setting website was monitored centrally, and for those participants who did not log on to access their risk score reports or for intervention participants who had not set goals two weeks from the time they were prompted by email, a study team member attempted to reach them by telephone to encourage them to access their results and set goals. After 4 weeks if results had not been accessed from the website, a printed MI and genetic risk score report was mailed to participants’ home.

Feasibility: Success at transmitting risk score information and health messages via electronic media (website, email, cell phone)
All participants were required to have an email access (including shared family email if they choose to use this email account) to be eligible for this study. The majority of participants (96%) had personal email access (Table 4). Most participants had no difficulty logging into website or viewing their results, although 23% reported having some technical problems with the website which inhibited the risk score report and health messages delivery.

4a-ii) Open vs. closed, web-based vs. face-to-face assessments:

All participants were followed up for a minimum of six months after randomization and repeat risk factor assessment was collected at the end of the study. End of study data were collected via face-to-face reassessment at the recruitment sites (n=238, (73.5%)) and by telephone or mail (n=86, (26.5%)). Repeat HbA1C and apoliporoteins A1 and B were also collected form participants who attended face-to-face reassessment visit.

4a-iii) Information giving during recruitment

Individuals were recruited from places of worship and community centers in Southwestern Ontario, Canada during the period from January 16, 2011 to January 29, 2012. Recruitment clinics were set-up in these “high-yield” locations at high yield times (following weekly ceremonies and scheduled activities) to maximize enrollment. The study team contacted community leaders in the recruitment locations to obtain permission to inform the congregation about the study, and this was done one to two weeks prior to the screening event.

Consent and Baseline Data Collection
Written informed consent, including consent to use of the health card number to facilitate future record linkage with health services databases, and to analyze DNA for genetic variants, was obtained from each participant. Information on risk factors including cholesterol status, diabetes, hypertension, current, former, and second hand exposure to tobacco smoke, diet, physical activity, sedentary behaviors, and psychosocial stress questions was collected. Stages of change information based on Prochaska’s model of change [29] was also obtained for diet, physical activity, sedentary behavior and smoking. Blood pressure (two measures 3 minutes apart using an automated OMRON device), body weight and height (to calculate BMI), waist and hip circumference, and body fat % using digital bioelectrical impedance scale were measured. A 30 mL non-fasting blood sample was collected from all participants, and was processed onsite within 2 hours of collection. The blood samples were analyzed for apolipoprotein A1 and B, HbA1C and the 9p21 (rs1333049) genotype. Previous studies have reported a minimal difference in apolipoprotiens’ levels when comparing fasting to non-fasting levels.[30]

4b) CONSORT: Settings and locations where the data were collected

Individuals were recruited from places of worship and community centers in Southwestern Ontario, Canada during the period from January 16, 2011 to January 29, 2012. Recruitment clinics were set-up in these “high-yield” locations at high yield times (following weekly ceremonies and scheduled activities) to maximize enrollment.

4b-i) Report if outcomes were (self-)assessed through online questionnaires

Using the information collected at the baseline visit, a MI risk report was generated for each participant using the INTERHEART risk score (IHRS) (Supplementary Material Table S1), which is a simple and valid risk factor scoring system developed and validated from the INTERHEART case-control study to assess MI risk in adult men and women.[31]
Approximately 4-6 weeks after screening visit was completed, participants were sent an email asking them to log onto the secured myOSCAR website (http://myoscar.org/), to access their risk score results. If they were eligible for randomization (based on study inclusion criteria), they were prompted to click on a button that took them to a web portal to be randomized to intervention or control (usual advice) groups using a computer generated algorithm in OSCAR (Open Source Clinical Applications & Resources).
Participants randomized to the intervention were prompted to choose a health goal on the website in the areas of i. healthy diet, ii. physical activity, iii. reducing sedentary behaviors, and iv. smoking cessation, and were prompted to update their goals weekly on the website. Participants then received health messages via email or text, tailored to their chosen health goal on a daily basis.
All participants were followed up for a minimum of six months after randomization and repeat risk factor assessment was collected at the end of the study. End of study data were collected via face-to-face reassessment at the recruitment sites (n=238, (73.5%)) and by telephone or mail (n=86, (26.5%)). Repeat HbA1C and apoliporoteins A1 and B were also collected form participants who attended face-to-face reassessment visit.

4b-ii) Report how institutional affiliations are displayed

5) CONSORT: Describe the interventions for each group with sufficient details to allow replication, including how and when they were actually administered

5-i) Mention names, credential, affiliations of the developers, sponsors, and owners

All participants were followed up for a minimum of six months after randomization and repeat risk factor assessment was collected at the end of the study. End of study data were collected via face-to-face reassessment at the recruitment sites (n=238, (73.5%)) and by telephone or mail (n=86, (26.5%)). Repeat HbA1C and apoliporoteins A1 and B were also collected form participants who attended face-to-face reassessment visit.
The study was approved by the McMaster/Hamilton Health Sciences research ethics board on June 3, 2009. The study was funded by a grant from the Heart and Stroke Foundation of Canada grant number NA# 6865.

5-ii) Describe the history/development process

The contents of the report were pre-tested in 2 focus groups conducted at a South Asian temple, and modified to the grade 5 reading level.
5-iii) Revisions and updating
This is the pilot phase of the trial. Proposed changes for the main trial are described:
Modifications to the SAHARA Large Trial Intervention: We have taken a number of steps to optimize the SAHARA intervention prior to testing its effective in MI risk reduction in a larger trial. These changes include: i. risk reports and randomization status will be emailed directly to participants, ii. the number of health goals participants can focus on has been reduced from 4 to 2, with only one being chosen at one time for a 6 month duration. iii. the duration of follow-up will be extended to 12 months with baseline, 6 months and 12 months face-to-face visits occurring, iv. increasing the frequency of in-person contacts to improve adherence to the intervention and interest in the program, v. health tips will be tailored to each participant based on the goal selected and their readiness to change, and vi. the frequency of messages will be reduced from daily to weekly and sent at a time of day chosen by participant.
5-iv) Quality assurance methods

5-v) Ensure replicability by publishing the source code, and/or providing screenshots/screen-capture video, and/or providing flowcharts of the algorithms used
Screen shots are provided in the appendices as well as the study website.
5-vi) Digital preservation
www.saharaproject.ca
5-vii) Access
Website usage and adherence to intervention “fidelity”
Participants usage of the goal setting website was monitored centrally, and for those participants who did not log on to access their risk score reports or for intervention participants who had not set goals two weeks from the time they were prompted by email, a study team member attempted to reach them by telephone to encourage them to access their results and set goals. After 4 weeks if results had not been accessed from the website, a printed MI and genetic risk score report was mailed to participants’ home.
5-viii) Mode of delivery, features/functionalities/components of the intervention and comparator, and the theoretical framework
Components for Intervention participants
•MyOSCAR Goal selection program: a tool which permits participants to select biweekly goals related to improving diet, increasing activity, decreasing smoking, and reducing sedentary behaviors
•Daily health messages – sent via email or text: messages provided tips on how to counter unhealthy habits and maintain healthy ones
•Biweekly reminders to pick a health goal and monitor progress on the goal
•Access to latest health information through personal MyOSCAR account
•Access to healthy living videos, such as yoga and other exercise regimens on www.saharaproject.ca
5-ix) Describe use parameters
Website usage and adherence to intervention “fidelity”
Participants usage of the goal setting website was monitored centrally.
5-x) Clarify the level of human involvement
Follow-up
All participants were followed up for a minimum of six months after randomization and repeat risk factor assessment was collected at the end of the study. End of study data were collected via face-to-face reassessment at the recruitment sites (n=238, (73.5%)) and by telephone or mail (n=86, (26.5%))
5-xi) Report any prompts/reminders used
Participants randomized to the intervention were prompted to choose a health goal on the website in the areas of i. healthy diet, ii. physical activity, iii. reducing sedentary behaviors, and iv. smoking cessation, and were prompted to update their goals weekly on the website.
5-xii) Describe any co-interventions (incl. training/support)
No co-intervention is used in this study.
6a) CONSORT: Completely defined pre-specified primary and secondary outcome measures, including how and when they were assessed
Pilot Study Outcome Measures
The two outcome measures of the pilot study included: 1) Feasibility of the intervention, defined by: a. success at transmitting risk score information and health messages via electronic media (website, email, cell phone), b. success at participants returning to use the website and set health goals, as this reflects the uptake of the intervention and helps to assess the effect of intervention on health behaviors, c. trend in the risk score change to indicate if the intervention leads to progressive health behavior change; and 2) Effectiveness and acceptability of risk score information was measured by: a. participants knowledge of their risk score over time, b. if this information induced positive behavior change, and c. participants’ satisfaction with the information received.
6a-i) Online questionnaires: describe if they were validated for online use and apply CHERRIES items to describe how the questionnaires were designed/deployed

6a-ii) Describe whether and how “use” (including intensity of use/dosage) was defined/measured/monitored

6a-iii) Describe whether, how, and when qualitative feedback from participants was obtained
Exit Survey
At the end of the pilot study, feedback from the study participants was obtained by asking all participants about their experiences of participating in the pilot study. The main feedback included: a. daily messages were too frequent which could potentially lead them to ignore the messages, b. phone calls to remind participants to login to the study website were too frequent, while others reported that there wasn’t enough in-person contact and would have liked to have a mid-program visit that with more face to face contact with the study team; and c. IHRS and GRS reports should be sent via email and remove the website login component. Most of the participants reported that participation in the SAHARA study was worthwhile for them. Supplementary Material Table S3 shows summary of the Exit Survey.
6b) CONSORT: Any changes to trial outcomes after the trial commenced, with reasons
This is a pilot study to inform the design of the larger trial.
7a) CONSORT: How sample size was determined
7a-i) Describe whether and how expected attrition was taken into account when calculating the sample size
This is a pilot study designed to address feasibility and specific objectives including recruitment to inform the larger trial design. No emphasis on sample size or statistical significance are placed in this manuscript.
7b) CONSORT: When applicable, explanation of any interim analyses and stopping guidelines
Not Applicable.
8a) CONSORT: Method used to generate the random allocation sequence

Randomization
Approximately 4-6 weeks after screening visit was completed, participants were sent an email asking them to log onto the secured myOSCAR website (http://myoscar.org/), to access their risk score results. If they were eligible for randomization (based on study inclusion criteria), they were prompted to click on a button that took them to a web portal to be randomized to intervention or control (usual advice) groups using a computer generated algorithm in OSCAR (Open Source Clinical Applications & Resources) - an open source software project launched by the Department of Family Medicine at McMaster University in Hamilton, Ontario, Canada in 2002, designed for the delivery of evidence-based resources and decision support at the point of care for both patients and providers (http://myoscar.org/).

8b) CONSORT: Type of randomisation; details of any restriction (such as blocking and block size)

No restrictions.

9) CONSORT: Mechanism used to implement the random allocation sequence (such as sequentially numbered containers), describing any steps taken to conceal the sequence until interventions were assigned

Approximately 4-6 weeks after screening visit was completed, participants were sent an email asking them to log onto the secured myOSCAR website (http://myoscar.org/), to access their risk score results. If they were eligible for randomization (based on study inclusion criteria), they were prompted to click on a button that took them to a web portal to be randomized to intervention or control (usual advice) groups using a computer generated algorithm in OSCAR (Open Source Clinical Applications & Resources).

10) CONSORT: Who generated the random allocation sequence, who enrolled participants, and who assigned participants to interventions

Approximately 4-6 weeks after screening visit was completed, participants were sent an email asking them to log onto the secured myOSCAR website (http://myoscar.org/), to access their risk score results. If they were eligible for randomization (based on study inclusion criteria), they were prompted to click on a button that took them to a web portal to be randomized to intervention or control (usual advice) groups using a computer generated algorithm in OSCAR (Open Source Clinical Applications & Resources)

11a) CONSORT: Blinding - If done, who was blinded after assignment to interventions (for example, participants, care providers, those assessing outcomes) and how

11a-i) Specify who was blinded, and who wasn't

laboratory procedures and analyses were blinded to the participants status.

11a-ii) Discuss e.g., whether participants knew which intervention was the "intervention of interest" and which one was the "comparator"

11b) CONSORT: If relevant, description of the similarity of interventions

Not applicable

12a) CONSORT: Statistical methods used to compare groups for primary and secondary outcomes

This is a pilot study. The results are presented in tabular form only.

12a-i) Imputation techniques to deal with attrition / missing values

Not used in this pilot study.

12b) CONSORT: Methods for additional analyses, such as subgroup analyses and adjusted analyses

not applicable

RESULTS

13a) CONSORT: For each group, the numbers of participants who were randomly assigned, received intended treatment, and were analysed for the primary outcome

A flow diagram of study participants is provided according to CONSORT. In addition all tables state the number of subjects per group.

13b) CONSORT: For each group, losses and exclusions after randomisation, together with reasons

A flow diagram of study participants is provided according to CONSORT. In addition all tables state the number of subjects per group.

13b-i) Attrition diagram

14a) CONSORT: Dates defining the periods of recruitment and follow-up

Individuals were recruited from places of worship and community centers in Southwestern Ontario, Canada during the period from January 16, 2011 to January 29, 2012.

14a-i) Indicate if critical "secular events" fell into the study period

Not applicable

14b) CONSORT: Why the trial ended or was stopped (early)

The pilot study has been completed.

15) CONSORT: A table showing baseline demographic and clinical characteristics for each group

Participants (n=412) were screened from 11 centers between January 2011 and January 2012, 41 were ineligible (5 had CVD, 23 had no email accounts, 13 were missing information required for the risk score, and 4 were eligible but not randomized due to a clerical error), leaving 367 participants randomized into the pilot study. Follow-up data collection occurred between October 28, 2011 and November 11 2012. The median time of follow-up is 280 days with interquartile range (IQR) of 252-319 days follow-up. As shown in Figure 1, forty three participants (12.6% and 10.8% of intervention and control group respectively) did not complete the follow-up (21 were not contactable and 22 participants withdrew from the study).
Demographic and Social Characteristics
Participants' characteristics are shown in Table 2.

15-i) Report demographics associated with digital divide issues

Demographic and Social Characteristics
Participants' characteristics are shown in Table 2. Briefly the mean age is 53.8 years, approximately half are women, and the majority of participants are immigrants to Canada. More than half reported speaking English at home, 88.7% received more than secondary school education and 69.2% are actively employed. More than 50% are vegetarian; few (1.2%) use or are exposed to tobacco, and approximately one-quarter (26.5%) engage in regular physical activity. Further, more than 32.0% are exposed to more than 2 hours of screen time per day.

16a) CONSORT: For each group, number of participants (denominator) included in each analysis and whether the analysis was by original assigned groups

16-i) Report multiple "denominators" and provide definitions

All tables include the number of participants in each group.

16-ii) Primary analysis should be intent-to-treat

17a) CONSORT: For each primary and secondary outcome, results for each group, and the estimated effect size and its precision (such as 95% confidence interval)

This is a pilot study only.

17a-i) Presentation of process outcomes such as metrics of use and intensity of use

Success at participants returning to use the website and set health goals
Participants use of the MyOSCAR-SAHARA website was monitored to determine how many participants logged on to the website to view their risk score reports and in the case of intervention participants, to set health goals. The login to the study MyOScar website was low for both groups (45.1% of intervention and 62.7% of control groups did not logon or used the website only once). The mean number of login attempts of the intervention group mean was 2.64 (SD 3.17, median 2.0) and control group was 1.63 (SD: 2.14, Median 1.0).

17b) CONSORT: For binary outcomes, presentation of both absolute and relative effect sizes is recommended

Not applicable

18) CONSORT: Results of any other analyses performed, including subgroup analyses and adjusted analyses, distinguishing pre-specified from exploratory

Pilot study only.

18-i) Subgroup analysis of comparing only users

19) CONSORT: All important harms or unintended effects in each group

Not Applicable

19-i) Include privacy breaches, technical problems

Feasibility: Success at transmitting risk score information and health messages via electronic media (website, email, cell phone)
All participants were required to have an email access (including shared family email if they choose to use this email account) to be eligible for this study. The majority of participants (96%) had personal email access (Table 4). Most participants had no difficulty logging into website or viewing their results, although 23% reported having some technical problems with the website which inhibited the risk score report and health messages delivery.
Table 4 Electronic Access and Reported Technical Difficulties

19-ii) Include qualitative feedback from participants or observations from staff/researchers

Exit Survey
At the end of the pilot study, feedback from the study participants was obtained by asking all participants about their experiences of participating in the pilot study. The main feedback included: a. daily messages were too frequent which could potentially lead them to ignore the messages, b. phone calls to remind participants to login to the study website were too frequent, while others reported that there wasn't enough in-person contact and would have liked to have a mid-program visit that with more face to face contact with the study team; and c. IHRS and GRS reports should be sent via email and remove the website login component. Most of the participants reported that participation in the SAHARA study was worthwhile for them. Supplementary Material Table S3 shows summary of the Exit Survey.

DISCUSSION

20) CONSORT: Trial limitations, addressing sources of potential bias, imprecision, multiplicity of analyses

20-i) Typical limitations in ehealth trials

We have taken a number of steps to optimize the SAHARA intervention prior to testing its effective in MI risk reduction in a larger trial. These changes include: i. risk reports and randomization status will be emailed directly to participants, ii. the number of health goals participants can focus on has been reduced from 4 to 2, with only one being chosen at one time for a 6 month duration. iii. the duration of follow-up will be extended to 12 months with baseline, 6 months and 12 months face-to-face visits occurring, iv. increasing the frequency of in-person contacts to improve adherence to the intervention and interest in the program, v. health tips will be tailored to each participant based on the goal selected and their readiness to change, and vi. the frequency of messages will be reduced from daily to weekly and sent at a time of day chosen by participant.

21) CONSORT: Generalisability (external validity, applicability) of the trial findings

21-i) Generalizability to other populations

This is a pilot study.

21-ii) Discuss if there were elements in the RCT that would be different in a routine application setting

22) CONSORT: Interpretation consistent with results, balancing benefits and harms, and considering other relevant evidence

22-i) Restate study questions and summarize the answers suggested by the data, starting with primary outcomes and process outcomes (use)

Discussion
We observed that a multimedia health behavior intervention is feasible in a South Asian population at risk for MI. While participants reported being motivated by receiving the risk score information, a number of features of the SAHARA intervention require optimization prior to assessing its effectiveness in MI risk factor reduction.

22-ii) Highlight unanswered new questions, suggest future research

Most participants had access to email, internet and text messages and had no difficulty receiving e-mail or text messages. However, our requirement of participants to proactively logon to the website to receive their risk reports, and to set goals was problematic with 23% of the study participants reporting technical difficulties. It is likely that this contributed to the low number of goals chosen over the course of the follow-up, and reduced the interventions potential impact on changing health behaviors. In addition, participants received the study messages either by email/text checked on a mobile device or emails checked on a fixed device. These different methods of receiving messages may have also impacted the uptake of the study intervention. The anticipated difference would be based on the fact that the mobile device message would be likely be received in real time or close to it, whereas the fixed device message might not be received immediately, although it may reach people when they're more ready to act on the information (i.e. they've specifically chosen to sit down at the computer, as compared to mobile when the email/text may arrive when the person is doing something else). In this study it is not known the impact of receiving messages via mobile or a fixed device on the intervention uptake and outcome.

Other information

23) CONSORT: Registration number and name of trial registry

ClinicalTrials.gov: NCT01577719

24) CONSORT: Where the full trial protocol can be accessed, if available

This is the pilot study of the trial.

25) CONSORT: Sources of funding and other support (such as supply of drugs), role of funders

The study was funded by a grant from the Heart and Stroke Foundation of Canada grant number NA# 6865.

X26-i) Comment on ethics committee approval

Methods
The study was approved by the McMaster/Hamilton Health Sciences research ethics board on June 3, 2009.

x26-ii) Outline informed consent procedures

Consent and Baseline Data Collection
Written informed consent, including consent to use of the health card number to facilitate future record linkage with health services databases, and to analyze DNA for genetic variants, was obtained from each participant.

X26-iii) Safety and security procedures

X27-i) State the relation of the study team towards the system being evaluated
Conflicts of Interest
None
